# Wet Adhesion and Adhesive Locomotion of Snails on Anti-Adhesive Non-Wetting Surfaces

**DOI:** 10.1371/journal.pone.0036983

**Published:** 2012-05-31

**Authors:** Neil J. Shirtcliffe, Glen McHale, Michael I. Newton

**Affiliations:** School of Science and Technology, Nottingham Trent University, Nottingham, United Kingdom; Massey University, New Zealand

## Abstract

Creating surfaces capable of resisting liquid-mediated adhesion is extremely difficult due to the strong capillary forces that exist between surfaces. Land snails use this to adhere to and traverse across almost any type of solid surface of any orientation (horizontal, vertical or inverted), texture (smooth, rough or granular) or wetting property (hydrophilic or hydrophobic) *via* a layer of mucus. However, the wetting properties that enable snails to generate strong temporary attachment and the effectiveness of this adhesive locomotion on modern super-slippy superhydrophobic surfaces are unclear. Here we report that snail adhesion overcomes a wide range of these microscale and nanoscale topographically structured non-stick surfaces. For the one surface which we found to be snail resistant, we show that the effect is correlated with the wetting response of the surface to a weak surfactant. Our results elucidate some critical wetting factors for the design of anti-adhesive and bio-adhesion resistant surfaces.

## Introduction

Snails adhere to surfaces by coating them with a thin (10–20 µm) layer of mucus [1]–[3], which is a complex mixture of polysaccharides and proteins [4]. To propel themselves over the mucus they use its non-linear properties to create pedal waves in a process known as adhesive locomotion [1]–[3], [5], [6]. This adhesive locomotion is one of the most energetically expensive, but effective, methods of locomotion known in biology [2]. The mucus snails' use allows them to adhere strongly and isolates them from the surface. This allows them to climb at any angle to the vertical, but does limit their size. Because their foot is attached at all times the danger of falling is much lower than with other methods of locomotion and they are able to attach to a greater variety of surfaces including the low energy non-adhesive polytetrafluoroethylene (PTFE) and the water-coated slippery hydrogels that represent the extremes of anti-adhesive non-slip materials. The adhesive advantages of this method of locomotion means it is being considered for small climbing robots, which are prone to falling if surfaces or surface conditions change [7]. The main disadvantages are the slow rate of movement, loss of water and high metabolic cost compared with other methods of locomotion [2].

One method of creating an anti-adhesive surface is to use microscale or nanoscale topographic features to amplify intrinsic non-wetting chemical properties of the surface. This can produce a superhydrophobic surface with a high droplet contact angle and small contact area, and low contact angle hysteresis [8], [9]. Droplets of water deposited on such a surface ball-up and roll-off so that they are often thought of as the ultimate type of slippery and non-stick surfaces [10]. The ability of a droplet to resist sliding from a surface as it is tilted is determined by the extent of its three-phase contact line and the contact angle hysteresis [11], [12]. This has recently been called shear hydrophobicity in contrast to tensile hydrophobicity, which is the ability for a droplet to resist being pulled away from a surface and which is determined by the receding contact angle rather than contact angle hysteresis [13]. The wetting properties of surfaces to snail mucus and the direction of forces on it is therefore likely to be of paramount importance in its adhesive properties.

## Results and Discussion

**Figure 1:**
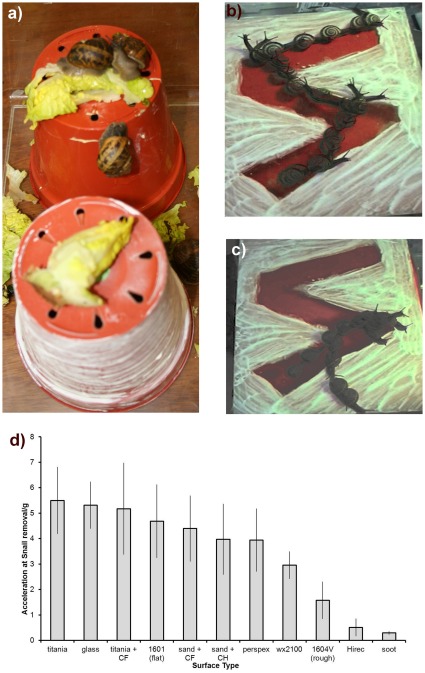
Snails cannot climb some surfaces. a) Snails have attacked the lettuce on one of the pots, but not on the other, even after 12 h. The upper pot was coated with Hirec 1440 superhydrophobic paint and the lower pot with 1604 V superhydrophobic coating. Overlaid sequence of a snail moving on a track bounded by a snail resistant superhydrophobic paint: b) track mounted vertically, and c) track inclined at a low angle. The figure was produced by selecting and compositing frames where the snail had moved forward by around one shell length.d) Acceleration in units of gravitational acceleration, g = 9.81 ms^−2^, required to remove snails from various surfaces rotated in the horizontal plane. Only the Hirec 1440 required less acceleration than 1 g.

To screen anti-adhesive properties of superhydrophobic surfaces, we first used a snail feeding experiment where we left lettuce leaves on two upturned plant pots, with different test coatings on their sides, within a snail-filled enclosure overnight. During these initial experiments it became clear that only one of the coatings used, Hirec 1440 (a superhydrophobic coating used to repel water on radar domes), was preventing the snails from climbing (Fig. 1a). Other coatings, although apparently similar when tested with water, were unable to prevent the snails from ascending over a 12 h period. A second superhydrophobic coating, Cytonix 1604 V, appeared to have a small effect, but did not prevent snail attack altogether.

To further examine the coating that appeared to frustrate the snails we used a snail track experiment consisting of a zigzag track on a sheet of polyacrylic created by painting around a path within which we desired to confine a snail. When the sheet was placed horizontally the snails were able to cross the painted area and did not deviate from their path when crossing the track boundary. This suggests that the effect of the coating was not related to toxicity, chemical dislike, loss of water or loose solid/powder particles coating the mucus and converting it to a solid-on-solid contact. Snails could attach to the top or bottom of this sheet when mounted horizontally, but when mounted vertically became detached almost immediately. When snails were placed on the track defined by the superhydrophobic paint and the sheet mounted vertically they moved and remained attached within the track, unless they could reach any other surface with their body (Fig. 1b and on-line Video S1). At intermediate angles the snails showed a limited preference for the track, remaining confined for a while and then escaping (Fig. 1c). Tracks created using other coatings neither presented problems to the adhesive performance of snails nor confined the snails to the track, although they appeared to be slowed by some of the coatings.

From these two types of experiments it was clear that a sliding force in the plane of the surface was most effective at removing a snail from a superhydrophobic surface as the snails could not climb a vertical surface but could hang on an inverted one. To assess the shear attachment strength of snails they were subjected to centrifugal forces by attaching them to a flat, horizontal plate that was spun at ever increasing rates until they slid off. Variance between runs on a single surface with a single snail was found to be as large as between different snails on that surface, so data from multiple snails was pooled. This is in line with previous work on the tribology of snails, which showed weak correlation between size and pull off force for terrestrial snails [14].

The coating that proved effective in confining snails to a defined track (Hirec 1440) required the smallest sliding force to remove a snail (Fig. 1d). Snails crawled off all surfaces if allowed a few minutes; no powder was observable on the snail's foot afterwards. The normal adhesive failure mode of the snails was in the mucus layer, which explains why most surfaces have similar removal forces in both this and a pull off experiment and why rough surfaces, with a thicker average mucus layer, require slightly less force [14]. Failure can also occur between the mucus and the surface as long as full contact between surface and mucus has not been achieved. This appears to occur on some of the superhydrophobic surfaces. The reduction in adhesive strength will be related to the fraction of the surface under the mucus that is air instead of solid. Visual inspection of the mucus left on the surfaces after colouring them with silver nitrate solution (1%w/w) revealed slime marks slightly larger than the snail foot on most surfaces. On the snail repellent surfaces predominantly ring shaped patterns were observed, suggesting that the edges of the snails' feet attached well, but their centres did not.

**Figure 2:**
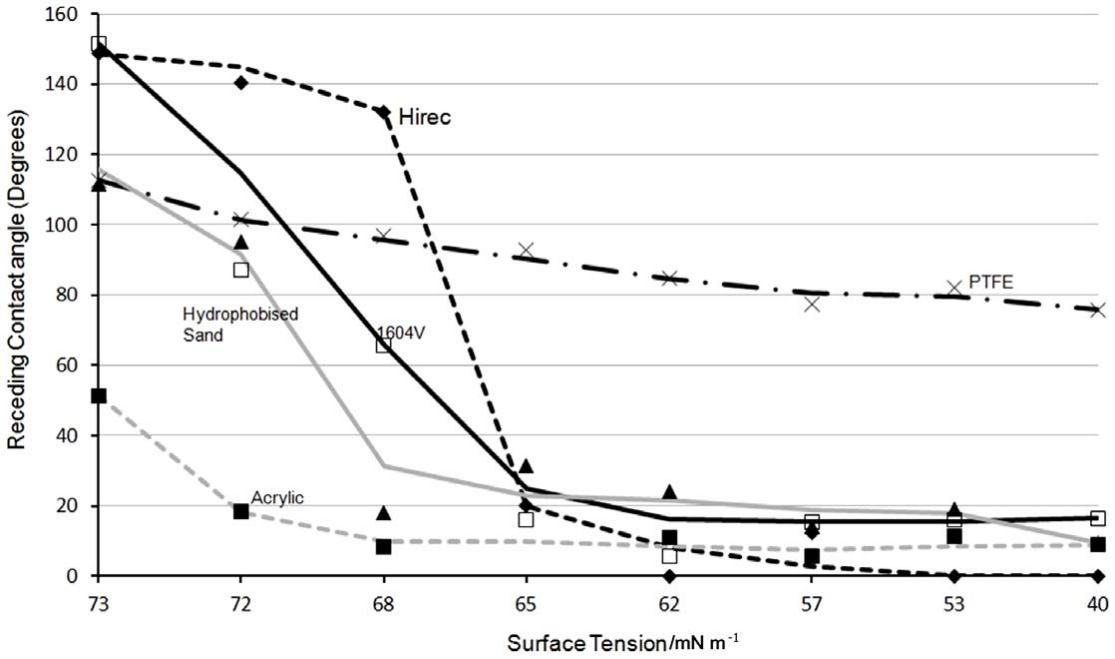
Plot of receding contact angles of sodium dodecylsulfate (SDS) solutions on different surfaces.

To characterise the wettability of the various surfaces on which snail adhesion was tested, we measured the advancing and receding contact angles of water, an oil (hexadecane), and an anionic surfactant (sodium dodecylsulfate) at various concentrations. No correlation was observed between the acceleration required to detach a snail in the centrifuge test and the advancing contact angle of any of the liquids. The oil fully wet the snail resistant surface. The differences between advancing and receding contact angle behaviour with surfactant concentration was the most revealing. Whilst advancing contact angle reduced progressively by around 20°–30° as surfactant concentration increased up to 100%, the receding contact angle dropped sharply to less than 20° on all surfaces except PTFE. However, the Hirec 1440 surface, which was able to prevent snails climbing, was able to maintain a high receding contact angle up to around 1 mM (1/8^th^ critical micelle concentration) before switching to a wetting state (Fig. 2). At this concentration the snail resistant Hirec 1440 had a higher receding contact angle than PTFE, but this switched at higher concentrations, indicating that some penetration into the roughness had occurred. Two of the other superhydrophobic surfaces, 1604 V and WX2100, which significantly reduced the acceleration needed to detach snails, also maintained higher receding contact angles at low surfactant concentrations, although they became wetted at lower surfactant concentration than Hirec 1440. Typically the receding angle on a superhydrophobic surface with surfactant was lower than that on an equivalent flat surface above a critical surfactant concentration. The composition of snail mucus has been studied [4], [15] and we hypothesize that the amphiphilic nature of the mucus is an important aspect of the adhesion on some surfaces.

The force needed to shed a liquid droplet from a surface is related to the unbalanced Young force integrated along the three-phase contact line. This results in a force that depends on the length of the three phase contact line, *p*, and the difference between the cosines of the advancing, θ*_A_*, and receding, θ*_R_*, contact angles, i.e. Force ∼ *pγ_LV_*(cosθ*_R_*-cosθ*_A_*), where γ*_LV_* is the liquid-vapour interfacial tension [16]. The high advancing contact angles typical of superhydrophobic surfaces reduce the extent of the three-phase contact line for a deposited droplet. However, the surface will only be slippery if the receding contact is also high so that contact angle hysteresis is low. In contrast, a non-superhydrophobic surface with a longer three-phase contact line and a lower advancing contact angle can be switched into a slippy surface by reducing the contact angle hysteresis as shown by 't Mannetje *et al*. using an electrowetting approach [17]. The determining role of the receding contact angle in liquid-based adhesion also extends to the work needed to pull a liquid from a surface, which has been reported to be determined by γ*_LV_*(1+cosθ*_R_*) [13], [18]. For a snail on a superhydrophobic surface this suggests that achieving liquid-mediated adhesion requires it to be able to reduce the receding contact angle of its mucus and to maintain a high level of contact angle hysteresis; these requirements can be met using surfactants. In particular, the ratio of net force *p*γ*_LV_*(cosθ*_R_*-cosθ*_A_*) for a normal hydrophobic horizontal surface with advancing and receding contact angles of θ*_A_* = 120° and, due to surfactant, θ*_R_* = 0°, and a snail resistant horizontal superhydrophobic surface with θ*_A_* = 150° and θ*_R_* = 120°as seen in fig. 2, is around 4. This is consistent with the results of the sliding removal test using centrifugal forces (Fig. 1d); although the perimeter of the foot of an 8 g snail is too short (80 mm) for the snail to adhere solely using this force.

The yield stress of garden snail mucus has been measured at 100–240 Pa [19]. This can be compared with the acceleration required to remove the snails. Using a mass of 8 g and a foot area of 800 mm^2^, an acceleration of 1 g would exert a stress of 98.1 Pa on the mucus. This shows that for the snail resistant Hirec surface the mucus is not shear thinned throughout during snail removal, although local yield at the contact points is likely. For normal surfaces the adhesion of snails up to around 4 g indicates that other mechanisms, such as suction, play a role; at least when the snail is stationary as the snails' mucus alone could only hold them against a little under 2.5 g.

Surfactants adsorb at liquid-solid and liquid-air interfaces, but are known to have a relatively weak effect on both the surface tension and advancing contact angle [20]. Only a small number of reports have been published on the effects of surfactants on the wetting of superhydrophobic surfaces [21]–[25]. The wetting of superhydrophobic surfaces by surfactants is not simple in that the addition of surfactant usually causes a transition from a bridging Cassie-Baxter type of wetting with sliding droplets to bridges that sit deeper into the surface, but do not fully penetrate it as they would in a Wenzel state [10]. With surfactants the stronger effect on receding contact angle on superhydrophobic surfaces, compared to advancing contact angle, is due to the creation of soap film bridges across the peaks of the roughness as the meniscus retracts [24], thus generating strong adhesion.

The size of gap that can be bridged by a soap film increases with surfactant concentration and so the transition to a low receding contact angle, and hence high wet adhesion, depends on a combination of the surface, the topography and the surfactant. As mucus is metabolically expensive to generate it seems likely that the supply will be low to limit its loss into the pores of the substrate. The balance between these requirements can be met by snail mucus using a weak bio-surfactant to achieve high advancing contact angles with low receding contact angles and hence a high adhesion and resistance to sliding. In our experiments, receding contact angles of a 1 mM solution of sodium dodecyl sulphate (1/8^th^ of the critical micelle concentration) were strikingly higher on the surface that snails could not climb and were low on those that they could. Outside a small range the differences between the surfaces became small. This suggests that the surface agent that snails use has a similar effect to this concentration of SDS.

The adhesive locomotive of land snails is remarkably effective across a vast range of surfaces, including modern micro- and nano-structured superhydrophobic ones, which shed droplets of water with ease. The key to their adhesive ability seems to be a precise control of the receding contact angle to increase contact angle hysteresis, using a weak surfactant. Understanding these new findings about the nature of adhesion in a biological system is important for understanding snail and other gastropod adhesive locomotion, for understanding properties of superhydrophobic surfaces, and for the design of adhesive and anti-adhesive surfaces of all types.

## Materials and Methods

*Cornu aspersum* (O.F. Müller 1774) (*helix aspersa*), from Blades Biologicals UK, and (*Cepaea nemoralis*) were used; snails of mass from 7.0 to 8.5 g were selected. For titania films Titanium isopropoxide (97% Aldrich), diethylene glycol (99% Aldrich) and ethanol (95% Fisher) were mixed in a volume ratio of 8.51∶2.4∶33.64, this was stirred for 1 h then a water and ethanol mix 0.45∶40 was prepared and added before stirring for a further 1 h and diluting to 25% in ethanol, glass substrates were dip coated then heated to 480°C at 2 C min^−1^ and maintained at that temperature for 2 h. Sieved sand (100–250 µm) was attached to surfaces using contact adhesive (No Nonsense, UK). To add a hydrocarbon finish samples were immersed in 1% octyltriethoxysilane (Aldrich deposition grade 98%) in toluene (99.5% Aldrich) for 24 h. To add a fluorocarbon finish, 20% v/v Extreme Wash in, Grangers UK was applied for 10 minutes, rinsed and then heated to 50 C for 24 h. Hirec 1440 (AT&T, Japan), Flutec LE12 (F2, UK) and Cytonix US 1601 and 1604 V were applied by brush and allowed to air dry for 1 day. Cytonix WX2100 was applied by aerosol. Advancing and receding angles were extracted from films (Krüss DSA10) of 5 µL drops on the surface being increased and decreased in volume at 40 µL min^−1^. Deionised water, hexadecane (>99% Aldrich) and sodium dodecyl sulfate (>99% Aldrich) in deionised water were used. Snails were placed into a covered glass tank and offered lettuce on the top of two differently coated inverted plastic plant pots 121 mm high. They were photographed after 30 min. and 10 h. The snail centrifuge was a modified spin coated, angular velocity was measured using an optical tachygraph, position and detachment time from video recordings.

## Supporting Information

Video S1 **Snails traversing a path bounded by a snail resistant superhydrophobic coating.**
(MOV)
